# Nutritional intervention during a teleprehabilitation pilot study in high-risk patients with colorectal cancer: adherence, motivators, and barriers

**DOI:** 10.1007/s00520-024-08915-3

**Published:** 2024-10-08

**Authors:** K. Beukers, R. F. W. Franssen, K. Beijaard, A. J. van de Wouw, R. C. Havermans, M. L. G. Janssen-Heijnen

**Affiliations:** 1 Department of Internal Medicine, VieCuri Medical Centre, Tegelseweg 210, 5912 BL Venlo, The Netherlands; 2 Department of Clinical Epidemiology, VieCuri Medical Centre, Venlo, The Netherlands; 3 Department of Epidemiology, GROW Research Institute for Oncology and Reproduction, Faculty of Health, Medicine and Life Sciences, Maastricht University, Maastricht, The Netherlands; 4 Department of Clinical Physical Therapy, VieCuri Medical Centre, Venlo, The Netherlands; 5 Department of Dietetics, VieCuri Medical Centre, Venlo, The Netherlands; 6 Department of Youth, Food, and Health, Faculty of Science and Engineering, Maastricht University, Maastricht, The Netherlands

**Keywords:** Colorectal cancer, Prehabilitation, Nutrition, Adherence

## Abstract

**Purpose:**

The preoperative period provides a window of opportunity to improve modifiable risk factors for treatment complications such as malnutrition, the so-called prehabilitation. Identifying factors related to adherence to nutritional interventions is essential for optimizing prehabilitation programs. The aim of this study is to evaluate a nutritional support module as part of a teleprehabilitation program in high-risk patients with colorectal cancer (CRC).

**Methods:**

A secondary analysis with a mixed method design of the nutritional support module of a pre-post teleprehabilitation pilot study was performed. Change in weight, complaints with intake, motivation, and subjective and objective adherence were evaluated.

**Results:**

Eleven patients were included. Subjectively, six patients (55%) were able to adhere to the nutritional advice. Despite that, nine of eleven (82%) patients experienced difficulties with the amount of food that was advised by the dietician. Six of eleven (55%) patients gained weight during the prehabilitation program. After prehabilitation, nine of eleven (82%) were able to reach 100% of their energy requirement and six of eleven (55%) were able to reach 100% of their protein requirement. Differences between patients in motivation and/or having complaints did not seem to be associated with protein and energy intake.

**Conclusion:**

This secondary analysis of a pilot study provides insights into understanding patients’ experiences with a nutritional support module as part of a teleprehabilitation program. With 82% of patients who were able to reach 100% of their energy requirement, teleprehabilitation seems to be feasible, while there is still room for improvement with respect to protein intake as only 55% of patients reached a sufficient intake.

## Introduction

Worldwide, colorectal cancer (CRC) is the third most common cancer and the second leading cause of cancer-related death. In 2018, approximately 1.8 million new patients were diagnosed with CRC, accounting globally for about 881,000 deaths [1]. Currently, surgical resection, with or without (neo)adjuvant chemo(radiation) therapy, is the primary treatment for non-metastatic CRC [2]. Over the last two decades, 5-year relative survival has increased substantially from 53% for patients diagnosed in the period 1991–1995 to 66% in the period 2011–2015 [3]. The occurrence of postoperative complications after surgical resection, however, remains high with an overall complication rate of approximately 30% [4].

Previous studies have shown that the risk for postoperative complications is higher among patients with low preoperative aerobic fitness and malnutrition [5–8]. To illustrate, Hu et al. showed a significant association of pre-operative hypoalbuminemia and overall complication rate with a three times higher risk [9]. In periods of stress (caused by, e.g., major abdominal surgery), protein needs are elevated, since there is an increased need for the synthesis of acute phase proteins and immune-related proteins for optimal recovery [10]. In case of insufficient provision of protein, independent of whether energy requirements are met, lean muscle mass can be broken down [8].

Efforts to improve postoperative outcomes have primarily focused on the intraoperative (i.e., laparoscopic surgery) and postoperative period (i.e., Enhanced Recovery After Surgery (ERAS)-protocol) [11, 12]. Since a few years, the preoperative period receives more attention, because this period provides a window of opportunity to improve modifiable risk factors preoperatively including malnutrition and physical fitness, the so-called prehabilitation [13]. A recent study by van Berkel et al. evaluated the effect of personalized prehabilitation (by a physical intervention but not a nutritional part) in high-risk patients with CRC undergoing elective surgery on reducing postoperative complications, showing a significantly reduced complication rate of 42.9% in the prehabilitation group compared to 72.4% in the usual care group [7]. A more holistic approach by a multimodal program with the combination of a physical and nutritional intervention may be synergetic, as suggested by a systematic review in 2018 [14].

Several recent studies suggest that prehabilitation promises health benefits and is feasible in patients with CRC [15–18]. However, these studies focused on physical fitness interventions in the preoperative period, leaving the importance of nutrition interventions relatively underexposed. One study explored the feasibility of a multimodal prehabilitation program encompassing physical exercise and protein-rich diet in patients with CRC. Despite information about the percentage of consumption, no information about factors affecting adherence to the diet was collected [19]. A scoping review by Gillis et al. showed that 34% of 110 included prehabilitation studies comprised a nutritional component. Of these, two-thirds did not monitor adherence to the nutrition intervention [20]. Clearly, there is a need for identifying and understanding patients’ reasons for adherence to nutritional interventions in order to further optimize prehabilitation programs [21]. Examining factors affecting adherence to nutritional interventions will provide essential knowledge for personalized nutritional programs that will ultimately reduce the risk of treatment complications. Since prehabilitation studies have only recently emerged, not to mention feasibility studies of prehabilitation, this study focused on a previous pilot study with inclusion of a representative population of high-risk patients with CRC.

The aim of this secondary analysis with a mixed method design is to evaluate change in weight as well as patient adherence, complaints, barriers, and/or motivators regarding a nutritional support module as part of a teleprehabilitation program in high-risk patients with CRC.

## Methods

### Study design and population

The present mixed method study is a secondary analysis of a pre-post tele-prehabilitation pilot study at a teaching hospital in the Netherlands [22] that was executed between July 2020 and September 2021. This pilot study consisted of 11 high-risk patients with non-metastatic CRC scheduled for elective resection with or without neoadjuvant chemotherapy. Patients were considered high-risk based on preoperative cardiopulmonary exercise testing (CPET). The full selection process and detailed inclusion criteria are described in the original study [22]. The current study focused on the nutritional intervention as part of the teleprehabilitation program.

### Study procedure

The teleprehabilitation program consisted of a physical fitness module [22] and a nutritional module based on national guidelines with optimization of basic nutritional requirement as well as sufficient protein intake (at least 1.5 g per kilogram per day). The protein intake should be spread throughout the day: 20–30 g of protein per main meal (in patient above 70 years: 25–30 g of protein per main meal), 20–30 g of proteins after physical training and a protein-rich snack before bedtime is recommended [22–24]. The exact requirement of protein and energy intake is based on the current weight before start of the teleprehabilitation program. Specific attention is given to the intake of vitamin D (food or supplementation) since this can positively influence muscle strength and muscle function. At last, attention is given to sufficient fluid (at least 1.5 to 2 l per day) with a correction for larger losses and fever (350 ml per degree) [22–24]. These nutritional advices were given by an experienced dietician with focus on oncological patients.

The program had a duration of 3 to 4 weeks, depending on the date of surgery. The surgery was planned according to standard care and was not delayed because of the prehabilitation program. After a face-to-face intake, telemonitoring was performed by follow-up meetings via weekly telephone sessions by a registered dietician executed as a semi-structured interview. The main outcome of this study was evaluated during the weekly follow-up sessions.

### Main outcome

The main outcome of this study was to evaluate patient experiences with the nutritional intervention as part of the teleprehabilitation program. The following items were evaluated as part of the evaluation: change in weight, subjective and objective adherence, complaints, barriers, and/or motivators. Adherence with the nutritional intervention was evaluated subjectively based on a self-report of dietary intake. Objective adherence was quantified by calculating protein and energy intake. As part of the semi-structured interview by the dietician, questions were asked about which motivators and/or barriers contributed to the adherence. In addition, any complaints with intake before start of the teleprehabilitation program were assessed. At last, patients were asked how motivated they were to participate in the nutritional intervention and which factors contributed to this motivation.

### Data collection and outcome measurements

During the face-to-face nutritional screening at baseline before start of the teleprehabilitation program, the following data was collected by a registered dietician (Table 1): age, sex, tumor location, cancer stage, type of chemotherapy, living situation (with/without partner), current weight, weight 1 month before start, weight 6 months before start, change in weight in the past 2 weeks, body mass index (BMI) in kg/m^2^, the patient-generated subjective global assessment short-form (PG-SGA-SF), the malnutrition universal screening tool (MUST), and serum albumin. By the use of a semi-structured interview at baseline, the following items were collected: complaints with intake (i.e., pain, feelings of obstruction, loss of appetite, nausea, digestion, taste and/or smell, stress due to diagnoses of cancer or general health), protein intake (calculated by Evry: a nutritional software program), energy intake (calculated by Evry), and motivation for participating in the nutritional program (by the use of a visual analog scale (VAS)).

**Table 1:** Data collection and outcome measurements registered over time (T0–T2)

	Data	Pre-prehabilitation	Prehabilitation	
		T0	T1	T2
Baseline characteristics	Age	X		
	Sex	X		
	Tumor location	X		
	Cancer stage	X		
	Chemotherapy regime	X		
	Living situation	X		
	Weight loss*	X		
	BMI (kg/m^2^)	X	X	X
	MUST score	X		
	PG-SGA-SF score	X		
	Albumin	X		
	Energy requirement	X		
	Protein requirement	X		
Evaluation of adherence	Actual weight	X	X	X
	Actual food intake	X	X	X
	Contributing factors		X	X
	Complaints with intake	X		
	Protein intake (% of requirement)	X		X
	Energy intake (% of requirement)	X	X	X
	Motivation (VAS)	X		

*BMI* body mass index, *MUST* malnutrition universal screening tool, *PG-SGA-SF* patient-generated subjective global assessment short-form, *VAS* visual analog scale. *Six months before diagnosis

Weekly follow-up telephone sessions were performed by the dietician. The telephone sessions were executed as a semi-structured interview and the following items were discussed and registered: weight, dietary intake (unchanged/increased/decreased), and evaluation of adherence to dietary advice (subjectively based on self-report of dietary intake). Protein and total energy intake were calculated at 2 (T1) and 4 weeks (T2) after start of the teleprehabilitation program.

Subjective information about adherence to nutritional advice and contributing factors were collected by evaluating the food diary of patients in a descriptive manner. The dietician collected these experiences and noted them by quotations of each patient. Before start of the teleprehabilitation program, the total requirement of protein and energy intake of each patient was calculated by the dietician. Based on the food diary of patients, the reached percentage of this protein and energy requirement was calculated before and after the prehabilitation program. Information about complaints with intake during the teleprehabilitation program were collected by using a standard questionnaire as part of the semi-structured interviews.

## Results

### Study selection

A total of eleven patients with non-metastatic CRC and scheduled for elective surgical resection were included. The median time between diagnosis (date of endoscopy) and surgery was 34 days (range 20–51 days) for patients who underwent surgery in the VieCuri Medical Centre (*n* = 10). One patient underwent surgery in another hospital due to required local surgical techniques. Due to short diagnosis-to-surgery intervals in five out of eleven patients, no T1 assessment could be performed in these patients. For that reason, only outcomes at T2 were included for analyses.

Baseline characteristics are presented in Table 2. The median age of the included patients was 73 years (range 57–85 years) and 54% was male. Three out of eleven patients had rectal cancer (27%) and one patient with rectal cancer received neo-adjuvant chemotherapy. Six patients had lost weight in the period of 6 months before diagnosis (range of 3–15kg). BMI ranged from 21 to 37kg/m^2^. Two out of eleven patients scored 2 points on the MUST and these patients also scored ≥ 9 points on the PG-SGA-SF. The mean level of serum albumin was 37 g/l (range 28–41 g/l).

**Table 2:** Baseline characteristics of included patients

	01	02	03	04	05	06	07	08	09	10	11
Age (years)	65	67	81	73	73	78	85	57	75	78	70
Sex (M/F)	Male	Female	Female	Male	Female	Male	Male	Male	Male	Female	Female
ASA-classification	3	3	3	2	4	3	3	-	2	3	3
Tumor location	Rectum	Ascending/transverse	Cecum	Cecum	Ascending	Rectum	Cecum	Rectum	Transverse	Cecum	Sigmoid
Cancer stage (I–IV)	III	I	I	II	I	I	III	I	II	III	I
Neoadjuvant CT	Cap	-	-	-	-	-	-	-	-	-	-
Living situation	Alone	Together	Alone	Together	Together	Alone	Together	Together	Together	Together	Alone
Change in weight (kg) *	− 6	− 15	=	=	=	=	=	− 7	− 3	− 7	− 9
BMI (kg/m^2^)	23.7	26.9	27.6	37.4	25.0	28.9	33.2	33.1	32.2	21.1	31.2
MUST	0	0	0	0	0	0	0	-	0	2	2
PG-SGA-SF	5	0	0	2	8	0	0	0	2	15	15
Serum albumin (g/l)	38	41	40	35	41	39	36	37	37	28	35

*ASA* American society anaesthesiologists *CT* chemotherapy, *Cap* capecitabin, *BMI* body mass index, *MUST* malnutrition universal screening tool, *PG-SGA-SF* patient-generated subjective global assessment short-form. * in the 6 months preceding the diagnosis of colon or rectal cancer

### Evaluation of adherence

Based on self-report by means of a food diary, six out of eleven patients were able to adhere to the nutritional advice. Contributing factors reported by patients were the following: clear information about the nutritional module, few adjustments to current diet, and large social network. Nine out of eleven patients experienced difficulties with the amount of food that was advised by the dietician. They indicated that they had to eat more than they were used to. This was the most frequently reported factor which negatively influenced the adherence. Other factors which negatively contributed to the adherence were described as follows: lack of clarity about protein-rich products, timing of meals with respect to traveling or sleep, and concerns about gaining weight during the program.

“I have to eat more than I’m used to.”

“...I don’t need an extra meal, I’m not hungry anymore.”

Six (patients 1, 5–7, 9, 10) out of eleven patients gained weight during the prehabilitation program, four (patients 2–4, 11) out of eleven patients stayed at the baseline weight, and only one patient lost weight (patient 8) (Table 3).

**Table 3:** Outcome measurements before and after the prehabilitation program per patient

ID	Subjective adherence	Change in weight (kg)*	Change in weight**	Complaints***	Energy intake (% of requirement)			Protein intake (% of requirement)			Motivation (VAS)	ND
			(kg)		T0	T2	Change (%)	T0	T2	Change (%)		
1	-	− 6	+ 2	+	142	144	+ 2 (+ 1%)	124	129	+ 4 (+ 3%)	7.5	+
2	+	− 15	=	-	93	139	+ 46 (+ 49%)	82	125	+ 43 (+ 52%)	7	-
3	-	=	=	-	97	123	+ 26 (+ 27%)	59	93	+ 34 (+ 58%)	8	-
4	-	=	=	-	80	103	+ 23 (+ 29%)	72	104	+ 32 (+ 44%)	8	-
5	+	=	+ 0.5	+	72	108	+ 36 (+ 50%)	60	95	+ 35 (+ 58%)	10	+
6	-	=	+ 1.5	+	73	128	+ 55 (+ 75%)	56	85	+ 29 (+ 52%)	7.5	-
7	+	=	+ 3	-	66	107	+ 41 (+ 62%)	54	87	+ 33 (+ 61%)	8	-
8	+	− 7	− 3	-	62	102	+ 40 (+ 65%)	50	108	+ 58 (+ 116%)	10	-
9	+	− 3	+ 1	-	58	75	+ 17 (+ 29%)	59	82	+ 23 (+ 39%)	8	-
10	+	− 7	+ 3	+	56	88	+ 32 (+ 57%)	63	130	+ 67 (+ 106%)	8	+
11	-	− 9	=	+	45	105	+ 60 (+ 133%)	44	104	+ 60 (+ 150%)	7	-
	Median				73	107	+ 34 (+ 47%)	59	104	+ 45 (+ 76%)		

*ND* nutritional drinks, *VAS* visual analog scale. *Six months before diagnosis of CRC. **During the prehabilitation program. *** + : complaints; -; no complaints

All patients increased their energy and protein intake during the prehabilitation program (Table 3). After prehabilitation, nine out of eleven (82%) were able to reach 100% of their energy requirement and six of eleven (55%) were able to reach 100% of their protein requirement. Three patients received medical nutritional drinks which also contains vitamins and minerals in addition to the protein-rich diet.

All patients were motivated for participating in the nutritional module in the prehabilitation program (mean VAS score of 8/10). Supporting factors for this motivation were described as follows: reducing risk of postoperative complications, disciplined character, pre-existent desire to lose weight, social support, and scientific interest.

“I am motivated for an optimal preparation for the operation, because I have a new girlfriend and want to live with her for at least another 10 years. She supports me.”

“…I am very disciplined to adjust my diet and exercise pattern. I wanted to lose weight anyway.”

Negatively contributing factors were described as follows: difficulty with timing (around holidays and Christmas), dependent on others for groceries, major dietary changes, dislike of dairy products, and stress due to diagnosis of cancer.

“I’m dependent on my children and neighbours for doing the groceries, so if I forget to write a product on the shopping list, it is not available.”

“…During the holidays I struggled with the time to do the physical exercises and also to follow the nutritional advices. I probably ate more than I should have.”

Four (patients 1, 5, 10, and 11) out of eleven patients experienced complaints with dietary intake. These complaints consisted of the following: fatigue, pain, feeling of obstruction, loss of appetite, and obstipation. One patient experienced diarrhea and loss of taste (this patient underwent neo-adjuvant chemotherapy before the prehabilitation program).

**Fig. 1:**
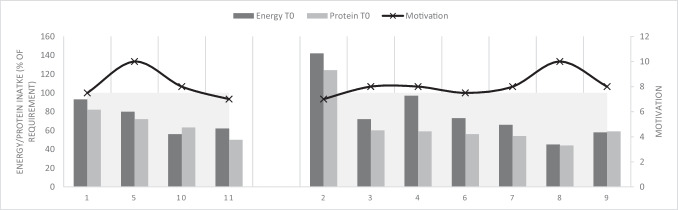
Energy/protein intake (% of requirement) and motivation (VAS score) per patient with complaints (patients 1, 5, 10, 11) and without complaints (patients 2–4, 6–9) before prehabilitation (T0)

**Fig. 2:**
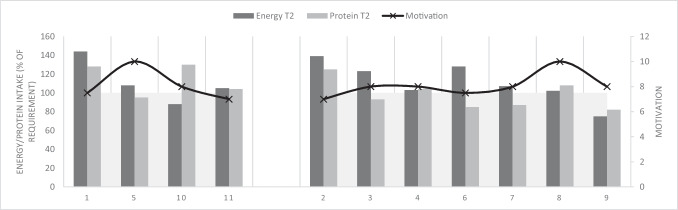
Energy/protein intake (% of requirement) and motivation (VAS score) per patient with complaints (patients 1, 5, 10, 11) and without complaints (patients 2–4, 6–9) after prehabilitation (T2)

In Fig. 1, the energy and protein intake (% of requirement) and motivation (VAS score) of patients with complaints (left-sided) and without complaints (right-sided) are shown before (T0) prehabilitation. In Fig. 2, the protein and energy intake (% of requirement) and motivation (VAS score) of patients with complaints (left-sided) and without complaints (right-sided) are shown after (T2) prehabilitation. After prehabilitation, six out of seven (86%) patients without complaints were able to reach 100% of their energy requirement and three out of seven (43%) patients without complaints were able to reach 100% of their protein requirement (Fig. 2). In patients with complaints, three out of four (75%) patients were able to reach 100% of their energy requirement after prehabilitation, and also, three out of four (75%) patients were able to reach 100% of their protein requirement after prehabilitation (Fig. 2).

The difference in motivation between patients related to both the protein and energy intake is shown in Figs. 1 and 2; patient number 8 with a maximum motivation score (VAS of 10) achieved + 65% in energy intake and + 116% in protein intake but another patient (patient number 11) with one of the lowest motivation scores (VAS of 7) achieved the most profit in both energy (+ 133%) and protein intake (+ 150%), despite complaints with intake.

## Discussion

The current mixed method study aimed to evaluate patient adherence and experiences with a nutritional support module as part of a teleprehabilitation program in high-risk patients with CRC. With 82% of patients who were able to reach 100% of their energy requirement, teleprehabilitation seems to be feasible, while there is still room for improvement with respect to protein intake as only 55% of patients reached a sufficient intake.

In the current study, six out of eleven patients (55%) reported they were able to adhere to the nutritional advice. Almost all patients (82%) experienced difficulty with the amount of food that was advised by the dietician; they indicated that they were advised to eat more than they were used to. Despite this fact, the majority of patients in this study were able to reach 100% of their protein and energy requirement. The observation that patients experienced difficulties in the amount of food they had to eat was consistent with a feasibility study with a multimodal prehabilitation program in older patients with CRC [19]. In the latter study, the prehabilitation program consisted of exercise training combined with six freshly prepared meals for 4 weeks.

Changes in weight measured before and after the teleprehabilitation program showed that six out of eleven gained weight, four out of eleven stayed at the baseline weight and only one patient lost weight. This latter patient stopped drinking alcohol during the prehabilitation program and improved his physical activity. Before entering the prehabilitation program, six out of eleven patients had lost weight to varying degrees. This should be considered when interpreting weight gain during the prehabilitation program since maintaining a stable weight throughout the program may be beneficial in these cases. Prehabilitation programs often focus on preventing (or reducing) preoperative weight loss [25, 26] but there is surprisingly little research aimed at examining the impact of weight change in the prehabilitation period on subsequent treatment tolerance. In addition, information about body composition in terms of lean body mass may be contributing since this assessment may even be more reflective of nutritional status than weight loss [27].

In our study, nine out of eleven (82%) of patients reached 100% of their energy requirement and six out of eleven (55%) patients reached 100% of their protein requirement after prehabilitation. These results are consistent with a prior multimodal prehabilitation study in nine patients with CRC with similar percentages of adherence [19]. The observation that in both studies, more patients were able to reach their energy requirements compared to their protein requirements may be explained by the fact that patients had to make more adjustments to their daily diet to reach sufficient protein intake than to reach sufficient energy intake. For example, in our study at baseline, patients already had an adequate level of energy intake (median of 73% of their requirement) compared to a median of 59% of protein requirement. If patients are at a lower level in their protein intake (median of 59% of their requirement in our study), they have to eat relatively more to meet 100% of their protein requirement. In line with the challenges in reaching sufficient protein intake, a Dutch study showed that institutionalized elderly have a mean protein intake of 0.8g/kg/day which means that these people have to increase their protein intake by more than one-third in order to reach a minimum of 1.5g/kg/day [28]. In some patients, medical nutritional drinks were used to increase energy and protein intake, but never, for example, a protein powder. The latter product may prove to be a better option in prehabilitation since protein powder can be added to a meal to increase its protein density without necessarily increasing the volume or even overall energy density of the meal [29]. At last, adequate education about the purpose of sufficient protein intake is essential since some patients in this study worried about gaining weight during the program. The fear of “eating too much” may hinder the patients in their intake.

Experiencing complaints with intake during the prehabilitation program (i.e., fatigue, pain, and loss of appetite) did not seem to have negatively influenced the energy and/or protein intake in our study. Interestingly, more patients with complaints were able to reach 100% of their protein intake compared to patients without complaints, though this finding is tentative considering the small numbers of patients in each group. In addition, differences in motivation between patients did not seem to be associated with the protein and energy intake in our study. However, major differences were not expected since the least motivated patient still scored a seven out of ten on the VAS assessing patient motivation.

This study has some limitations. First, the sample size of this study is small with only eleven patients, so selection bias may exist. However, the optimal minimum regarding the sample size of feasibility studies is currently unclear and the small sample in this study provided the opportunity to perform an in-depth analysis. Moreover, the included eleven patients seem to be representative of a larger population of high-risk patients with CRC since the characteristics are comparable with characteristics in a larger single-blinded randomized controlled trial in the Netherlands [7]. Second, the short diagnosis-to-surgery interval remains a major hurdle for optimal implementation of an adequate nutritional intervention in the teleprehabilitation program. For example, in five out of eleven patients in this study, no mid-term (T1) assessment could be performed. The short interval periods are mainly due to logistical reasons (i.e., delayed final diagnoses and/or surgical planning) and subsequent pressure with current waiting time targets. Extending the time interval between diagnosis and surgery would enable adopting an adequate prehabilitation program but it remains unclear whether this is safe in terms of survival. In fact, it is unclear if there is an optimal diagnosis-to-treatment interval. A recent systematic review by Franssen et al. demonstrated that current short diagnosis-to-treatment time limitations are not supported by literature [30]. In addition, time constraints with respect to inclusion of patients after diagnosis remain challenging as there are often many assessments (i.e., imaging in the context of staging) and procedures (multidisciplinary meetings) before a patient is educated about the surgery and the possibility of teleprehabilitation [22]. Perhaps, nutritional screening and optimization should at first contact with the hospital (i.e., after endoscopy) in order not to lose precious time. Third, the assessment of having complaints with intake and motivation was performed only at the beginning of the prehabilitation program. It would have been more reliable if these assessments had been repeated during the period of prehabilitation in order to evaluate a possible change over time. At last, dietary intake was evaluated subjectively based on a self-report. It would have been more accurate to measure dietary intake by, for example, a structured process using 24 h as developed by the National Cancer Institute (NCI) [31, 32]. However, since this was a secondary analysis of pre-existent data, we were restricted to the methods for data collection that had been used in the original study.

The results of this study could contribute to the development of prehabilitation programs by a better understanding of patients’ experiences and preferences with a nutritional intervention. For example, this study demonstrated that the amount of food intake is challenging for the majority of patients so this issue requires more attention, i.e., by using protein powders instead of increasing the amount of meals, as noted above. More evidence with respect to (optimal) weight change and body composition during the prehabilitation program is necessary. In addition, this study emphasizes the need for adequate education about the purpose and expectations of the nutritional support module since, for example, some patients worried about gaining weight during the program. At last, it would be useful to extend the current findings by examining other factors, such as influence of social support, that might impact the adherence of nutritional interventions during prehabilitation.

In conclusion, this study showed that a nutritional support module as part of a teleprehabilitation program is feasible with the majority of patients who reached a sufficient intake of both protein and energy intake. Understanding the patients’ perspective is crucial since this enables tailoring of the nutritional intervention to the preferences of the patient in order to optimize prehabilitation programs with a reduced incidence of complications as a result.

## Data Availability

Data is provided within the manuscript.
